# Changes in the Default Mode Network and Recovery of Impaired Consciousness in Traumatic Brain Injury: A Case Series

**DOI:** 10.3390/diagnostics16132026

**Published:** 2026-06-29

**Authors:** Sung Ho Jang, Dong Hyun Byun

**Affiliations:** 1 Department of Physical Medicine and Rehabilitation, College of Medicine, Yeungnam University, Namku, Daegu 42415, Republic of Korea; strokerehab@hanmail.net; 2 Department of Special Creative Convergence, College of Rehabilitation Sciences, Daegu University, Jillyang-eup, Gyeongsan-si 38453, Republic of Korea

**Keywords:** medial prefrontal cortex, posterior cingulate cortex, default mode network, impaired consciousness, diffusion tensor imaging, traumatic brain injury

## Abstract

**Background and Clinical Significance**: We report on three patients with traumatic brain injury (TBI) who showed improvement of impaired consciousness and changes in the default mode network (DMN), which was demonstrated on follow-up diffusion tensor tractographies (DTTs). **Case Presentation**: Three TBI patients underwent comprehensive rehabilitation during their first and second evaluations. Their Coma Recovery Scale-Revised (CRS-R) scores were obtained, and DTT was performed at 98.7 ± 22.0 days (first) and subsequently at 216.0 ± 78.1 (second) after onset. Compared with the first evaluation, the CRS-R scores increased by 12, 8, and 10 points on the second evaluation of the three patients. Although not reconstructed on the results of the first DTT, the second DTT results showed reconstruction of the medial prefrontal cortex (mPFC)–posterior cingulate cortex (PCC) DMN in patients 1 and 2. Regarding the continuously reconstructed DMNs (both mPFC-PCC and mPFC–precuneus), structural thickenings were generally observed in patients 1 and 3; however, narrowing was observed on the right mPFC-PCC DMN of patient 3. On the second DTT, all fractional anisotropy values of the reconstructed DMNs increased in patients 1 and 3, whereas all mean diffusivity values of the reconstructed DMNs decreased. Except for the right mPFC-PCC DMN in patient 3, all tract volume values of the reconstructed DMNs increased on the second DTT. **Conclusions**: DTT demonstrated changes in the mPFC-PCC/precuneus DMN concurrent with the improvement of impaired consciousness in three patients. In conclusion, recovery of the mPFC-PCC/precuneus DMN is closely associated with improvement of impaired consciousness in TBI patients.

## 1. Introduction

Traumatic brain injury (TBI) is an injury to the brain caused by an external force, and it has been reported that 12.1% of people will experience TBI during their lifetime [1]. Among the various neurological manifestations of TBI, impaired consciousness is common (37.7%) and can be reversible or irreversible, resulting in patient suffering and increased material costs to society [2]. Therefore, elucidation of neural changes associated with recovery of impaired consciousness in TBI patients is important in establishing treatment strategies for patients with impaired consciousness following TBI.

The neural network for consciousness comprises various neural connections such as the default mode network (DMN), the ascending reticular activating system, thalamo-cortical connectivity, temporoparietal connectivity, fronto-parietal connectivity, and cortical effective connectivity [3,4,5,6,7,8,9,10]. The DMN is a specific, anatomically defined brain system preferentially active when individuals are not focused on the external environment [5]. Four areas of the DMN have been considered as important in the control of consciousness: the medial prefrontal cortex (mPFC), the temporoparietal junction, the posterior cingulate cortex (PCC), and the precuneus [6,7,8]. Among these network connections, those between the mPFC and the PCC/precuneus are reported to be involved in internal awareness and consciousness [6,7,8,9].

Because the DMN is not fully discriminated from adjacent neural structures when using conventional brain computed tomography or magnetic resonance imaging, the precise estimation of the DMN has been limited in the live human brain. However, the recent development of diffusion tensor tractography (DTT), derived from diffusion tensor imaging (DTI), enables such estimation by allowing three-dimensional reconstruction of neural tracts, and DTT has the further advantage of allowing the observer to detect changes in neural tracts by examining tract configuration and quantification of tract parameters [11,12,13,14].

Previous studies have used DTI to demonstrate relationships between the DMN and various clinical manifestations, such as cognitive impairment, anxiety, and depression [15,16,17,18,19,20]. However, while one previous study reported an association between DMN status and consciousness, no study has demonstrated longitudinal changes in the DMN during the recovery of impaired consciousness. Furthermore, DTT has not been used previously to detect these structural changes in the DMN during the recovery process.

In the current study, we report on three TBI patients who showed improved consciousness in concert with changes in their DMN, with changes demonstrated by using follow-up DTTs. Through this study, we attempted to demonstrate that longitudinal tracking of DTT parameters can provide direct anatomical evidence of structural neuroplasticity, suggesting that it can be a useful imaging biomarker for monitoring consciousness recovery in severe TBI.

## 2. Case Report

### 2.1. Methods

#### 2.1.1. Subjects

Three patients (three right-handed men; mean age, 51.66 years; age range, 26–81 years) who showed improvement of impaired consciousness between their first and second evaluations were recruited for this study according to the following inclusion criteria: (1) first-ever TBI; (2) DTI was performed twice with a minimum of one month between first and second evaluations; (3) no previous history of psychiatric, neurological, or physical illness, or TBI before the current trauma; and (4) Coma Recovery Scale-Revised (CRS-R; full score, 23) score increase between first and second evaluations [21]. The institutional review board of the university hospital approved the study protocol, and the guardians of the patients in this study provided signed informed consent.

#### 2.1.2. Diffusion Tensor Imaging

The DTI data were acquired twice: first DTI at a mean duration from TBI onset of 98.67 ± 21.94 days; second DTI at a mean duration from TBI onset of 216.00 ± 78.08 days (117.33 ± 69.53 days between the first and second evaluation). Imaging was accomplished using a 6-channel head coil on a 1.5 T Philips Gyroscan Intera magnetic resonance imaging scanner (Philips, Best, The Netherlands) with single-shot spin-echo planar imaging. For each of the 32 non-collinear diffusion sensitizing gradients, 63 contiguous slices were acquired parallel to the anterior commissure–posterior commissure line. Imaging parameters were as follows: acquisition matrix = 96 × 96, reconstructed to matrix = 192 × 192, field of view = 240 mm × 240 mm, repetition time = 10,398 ms, echo time = 72 ms, parallel imaging reduction factor (SENSE factor) = 2, echo planar imaging factor = 59, b = 1000 s/mm^2^, number of excitations = 1, and slice thickness = 2.5 mm.

#### 2.1.3. Probabilistic Fiber Tracking

Analytical tools within the Oxford Centre for Functional Magnetic Resonance Imaging of the Brain (FMRIB) software(version 6.0.6) library were used to analyze diffusion-weighted imaging data. Affine multi-scale two-dimensional registration was used to correct head motion effects and image distortion due to eddy currents. A probabilistic tractography method based on a multi-fiber model was used for fiber tracking and was applied in the current study by utilizing tractography routines implemented in FMRIB Diffusion software(version 6.0.6) (0.5 mm step lengths, 5000 streamline samples, curvature threshold = 0.2) [22,23,24].

**Figure 1:**
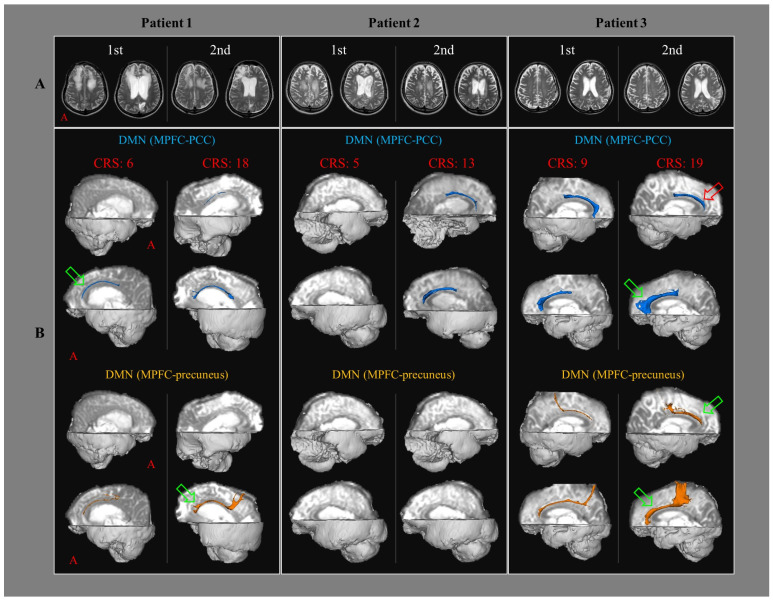
(**A**) The first and second T2-weighted brain magnetic resonance images of three patients of traumatic brain injury. (**B**) Diffusion tensor tractography (DTT) for the medial prefrontal cortex (mPFC)–posterior cingulate cortex (PCC) default mode network (DMN) and the mPFC–precuneus DMN. On the second DTT, reconstruction of the mPFC-PCC DMN is observed on the right side of patient 1 and both sides of patient 2, which were not reconstructed on the first DTT; in addition, thickening of the left side of patient 3 is observed (green arrows). However, narrowing is observed on the right side of patient 3 (red arrow). Thickening of the mPFC–precuneus DMN is observed on the left side of patient 1 and both sides of patient 3 (green arrows).

For analyses of the neural networks between the mPFC and the PCC/precuneus, a seed region of interest (ROI) was placed on the mPFC (Brodmann’s areas 14, 24, 25, and 32). The superior boundary of the ROI was the cingulate sulcus, while the medial boundary was the midline between the right and left hemispheres, and the lateral boundary was a line 11.25 mm lateral from the midline [25]. Target ROIs were placed on the PCC and the precuneus (Figure 1A) [7].

From the 5000 samples generated for each seed voxel, results for each contact were visualized based on the threshold and weightings of the tract probability analysis, which was set at a minimum of two streamlines through each voxel. The fractional anisotropy (FA), mean diffusivity (MD), and tract volume (TV) values for the neural networks between the mPFC and the PCC/precuneus were determined for both hemispheres.

## 3. Results

### 3.1. Patient 1

A 26-year-old male patient was diagnosed with traumatic subarachnoid hemorrhage (T-SAH), traumatic epidural hemorrhage, and traumatic intracerebral hemorrhage after falling on stairs (Table 1; Figure 1A).

He underwent comprehensive rehabilitation during the four months following onset. The patient’s consciousness increased 12 points on CRS-R from a CRS-R score of 6 (auditory function scale: 0; visual function scale: 1; motor function scale: 2; oromotor/verbal function scale: 1; communication scale: 0; arousal scale: 2) at four months after onset (first evaluation) to a CRS-R score of 18 (auditory function scale: 3; visual function scale: 4; motor function scale: 6; oromotor/verbal function scale: 2; communication scale: 1; arousal scale: 2) at ten months after onset (second evaluation). The right mPFC-PCC DMN at first evaluation DTT and the right mPFC–precuneus DMN on the first and second evaluation DTTs were not reconstructed. However, the right mPFC-PCC DMN on the second DTT and the left mPFC-PCC DMN and the mPFC–precuneus DMN were reconstructed on both the first and second DTTs (Figure 1B). Compared to the results from the first DTT evaluation, the FA and TV values increased, whereas the MD values of the left mPFC-PCC DMN and the mPFC–precuneus DMN decreased on second DTT (Table 2).

### 3.2. Patient 2

A female patient (48-year-old) was diagnosed with T-SAH and intraventricular hemorrhage after falling from a 5th-floor location (Table 1; Figure 1A). The patient started comprehensive rehabilitation at two months after onset. The patient’s CRS-R score increased by 8 points between the first evaluation (2 months after onset; CRS-R: 5 [auditory function scale: 0; visual function scale: 1; motor function scale: 2; oromotor/verbal function scale: 1; communication scale: 0; arousal scale: 1]) and the second evaluation (six months after onset, CRS-R: 13 [auditory function scale: 3; visual function scale: 3; motor function scale: 3; oromotor/verbal function scale: 1; communication scale: 0; arousal scale: 3]). In both hemispheres, the mPFC-PCC DMN was not reconstructed on the first DTT evaluation; however, it was reconstructed in both hemispheres on the second DTT. In contrast, the mPFC–precuneus DMN was not reconstructed in either hemisphere on both the first and second DTTs (Figure 1B).

### 3.3. Patient 3

An 81-year-old male patient was diagnosed with T-SAH, traumatic subdural hemorrhage, and traumatic intracerebral hemorrhage (Table 1). He underwent comprehensive rehabilitation beginning three months after onset. His second CRS-R score was 10 points higher than that on the first CRS-R. His first evaluation CRS-R score was 9 (3 months after onset: auditory function scale: 2; visual function scale: 1; motor function scale: 2; oromotor/verbal function scale: 1; communication scale: 1; arousal scale: 2), while the second evaluation CRS-R score was 19 (5 months after onset: auditory function scale: 4; visual function scale: 5; motor function scale: 5; oromotor/verbal function scale: 2; communication scale: 1; arousal scale: 2). The mPFC-PCC DMN and the mPFC–precuneus DMN were reconstructed in both hemispheres on both the first and second DTTs. Compared to the first DTT results, there were increments in the FA and TV values and a decrement in the MD value on the second DTT for both the mPFC–precuneus DMNs and the mPFC-PCC DMNs, except the right mPFC-PCC DMN had a lower TV value on the second DTT evaluation (Figure 1B).

## 4. Discussion

In this study, we used DTT to evaluate changes in the DMN (a neural network between the mPFC and the PCC/precuneus) during recovery from impaired consciousness in three patients with TBIs. We observed the following changes: (1) Configurational changes on the second DTT compared with that on the first DTT; i.e., reconstruction of the mPFC-PCC DMN was observed on the right side of patient 1 and both sides of patient 2, which were non-reconstructed on the first DTT. There was thickening of the left side mPFC-PCC DMN of patient 3; however, narrowing was observed on the right side mPFC-PCC DMN of patient 3. Regarding the mPFC–precuneus DMN, thickening was observed on the left side of patient 1 and both sides of patient 3. (2) Changes in DTT parameters on the second DTT compared with those from the first DTT; i.e., increases in all FA values of the reconstructed DMNs in patients 1 and 3, decreases in all MD values of the reconstructed DMNs, and increases in all TV values of the reconstructed DMNs except for the right mPFC-PCC DMN in the patient 3. These changes in the DTT parameters indicate that the following changes occurred in the DMNs: the increased FA value is indicative of increased directionality, the decreased MD value indicates a decreased magnitude of water diffusion, while the increased TV value indicates an increase in neural fibers [26,27]. Thus, our results suggest that recovery of the injured mPFC-PCC/precuneus DMNs and the improvements to the impaired consciousness in these three patients with TBI occurred concurrently.

Many studies have reported relationships between clinical manifestations related to cognition and the state of the DMN in various brain pathologies, including Alzheimer’s disease, attention deficit hyperactivity disorder, and depression, based on results from positron emission tomography and resting functional MRI (fMRI) [28,29,30,31,32,33]. Likewise, many studies have also reported on the state of the DMN according to the state of consciousness in patients with hypoxic–ischemic brain injury, stroke, encephalitis, and TBI by using resting fMRI [34,35,36,37,38,39]. Moreover, there have been two fMRI-based studies that have demonstrated that the state of the DMN during the acute stage of TBI and cardiac arrest had prognostics predictability for impaired consciousness during the chronic stage [40,41]. While previous functional studies (fMRI/PET) link DMN disruption to impaired consciousness in TBI, the structural basis for network reorganization during recovery remains unclear. Our longitudinal DTT findings provide direct structural evidence through the observed reconstruction of the mPFC-PCC/precuneus tracts, which may anatomically underpin the functional DMN recovery reported in prior literature.

Since the development of DTI, several studies have demonstrated relationships between clinical manifestations, such as cognitive impairment, anxiety, and depression, and the state of the DMN in preterm infants, autism, encephalopathy, and TBI [15,16,17,18,19,20]. Regarding impaired consciousness, one study used DTT to reveal a relationship between impaired consciousness and the state of the DMN [6]. In 2102, Fernandez-Espejo et al. reported changes in the consciousness state according to the DMN state in 52 patients with impaired consciousness following TBI compared to non-TBI subjects; the TBI occurrences (19 vegetative state, 27 minimally conscious state, six extended mini) were not identified by a specific pathology [6]. The authors classified the DMN results into connectivities between the right and left temporoparietal junction (TPJ), PCC/precuneus–mPFC, mPFC-TPJ, PCC/precuneus–TPJ, PCC/precuneus–thalamus, MPFC–thalamus, and TPJ–thalamus, and found that all of areas of the DMN, except for the mPFC–thalamus and TPJ–thalamus, showed decreased connectivity in TBI patients compared with normal control subjects. Moreover, the FA value of the DMN between PCC/precuneus–thalamus and PCC/precuneus–TPJ showed a correlation with the CRS-R score [6]. Based on those previous studies, to the best of our knowledge, this is the first study to use DTT to demonstrate changes in the DMN (mPFC-PCC/precuneus) occurring in concert with impaired consciousness improvements in patients with TBI. However, several limitations of this study should be considered. First, DTT analysis may underestimate or overestimate the fiber tracts in regions of fiber complexity or crossing fibers [42]. Second, because it is a case series based on only three patients, the results cannot be generalized to the broader TBI population. Therefore, the findings of the present study must be regarded strictly as exploratory and hypothesis-generating rather than confirmatory. Third, the observed association between the reconstruction or thickening of specific DMN pathways and the improvement in CRS-R scores demonstrates a concomitant relationship, but does not definitively establish causality. To address these limitations, future multicenter studies with larger cohorts are necessary to establish generalizability. Furthermore, integrating multimodal neuroimaging (e.g., DTT with fMRI/PET) is required to validate the functional correlates of structural DMN reorganization during clinical recovery.

In conclusion, by using DTT, we demonstrated changes in the mPFC-PCC/precuneus DMN that occurred concomitantly with the improvement of impaired consciousness in three patients with TBI. Despite the limitation of a small number of subjects, we believe that longitudinal DTT is a useful tool for elucidating the structural neuroplasticity of the DMN. Unlike previous functional neuroimaging studies, DTT has the advantage of demonstrating the actual structural reorganization of neural tracts accompanying clinical recovery. Therefore, we suggest that tracking DTT parameters can be a useful structural biomarker for monitoring the recovery of severe TBI, and this could be helpful in establishing future neurorehabilitation strategies.

## Figures and Tables

**Table 1:** Demographic and clinical characteristics of three patients.

	Patient 1	Patient 2	Patient 3
Sex/age (year)	M/26	F/48	M/81
Lesion	T-SAH, T-EDH + T-ICH	T-SAH + T-IVH	T-SAH + T-SDH + T-ICH
First DTI duration from onset (days)	116	74	106
Second DTI duration from onset (days)	304	189	155
Duration between first and second DTI (days)	188	115	49
Change in CRS-R score	+12 (6 to 18)	+8 (5 to 13)	+10 (9 to 19)

M: male; F: female; T-SAH: traumatic subarachnoid hemorrhage; T-EDH: traumatic epidural hemorrhage; T-ICH: traumatic intracerebral hemorrhage; T-IVH: traumatic intraventricular hemorrhage; T-SDH: traumatic subdural hemorrhage; DTI: diffusion tensor imaging; CRS-R: Coma Recovery Scale-Revised.

**Table 2:** Comparison of changes in diffusion tensor tractography parameters for the default mode network and the Coma Recovery Scale-Revised scores of three patients.

DMN		Patient 1		Patient 2		Patient 3	
		1st	2nd	1st	2nd	1st	2nd
mPFC-PCC	FA	-/0.33	0.31/0.34	-/-	0.29/0.26	0.32/0.32	0.33/0.34
	MD	-/1.03	1.11/0.97	-/-	0.98/0.93	1.98/1.58	0.85/0.91
	TV	-/235.00	78.00 /413.00	-/-	291.00 /563.00	776.00 /1033.00	671.00 /1595.00
mPFC–Precuneus	FA	-/0.33	-/0.36	-/-	-/-	0.30/0.29	0.35/0.38
	MD	-/0.93	-/0.90	-/-	-/-	0.85/0.98	0.80/0.83
	TV	-/207.00	-/676.00	-/-	-/-	268.00 /502.00	897.00 /1194.00
CRS-R		6	18	5	13	9	19

DMN: default mode network; mPFC: medial prefrontal cortex; PCC: posterior cingulate cortex; FA: fractional anisotropy; MD: mean diffusivity; TV: track volume; CRS-R: Coma Recovery Scale-Revised.

## Data Availability

The data presented in this study are available on request from the corresponding author. Data available on request due to privacy/ethical restrictions.
